# Education, work, and the production of vulnerability: experiences of women with visual impairments in China

**DOI:** 10.3389/fsoc.2026.1740235

**Published:** 2026-03-02

**Authors:** Minjie Chen

**Affiliations:** School of Sociology and Social Policy, University of Nottingham, Nottingham, United Kingdom

**Keywords:** disability in China, education, employment, family life, vulnerability, women with visual impairments

## Abstract

Women with visual impairments often experience compounded disadvantages at the intersection of gender and disability, yet research examining these dynamics in specific social and institutional contexts remains limited. Drawing on qualitative data from China, this study explores how barriers in education and employment create vulnerability and constrain autonomy among women with visual impairments. Seven women aged 25–40 with work experience participated in semi-structured interviews conducted remotely. The findings indicate that early vocationalisation, restricted access to tertiary education, and channelled career pathways contributed to structural precarity and socially produced vulnerability, while gendered expectations further constrained agency. At the same time, participants described strategies for navigating constraints and exercising agency within existing limitations. These findings underscore the importance of context-sensitive approaches to inclusive education, employment diversification, and social recognition in efforts to enhance protection, autonomy, and empowerment for women with visual impairments within specific institutional settings in China.

## 1 Introduction

Vulnerability arises when individuals lack the capacity to respond to threats that compromise their well-being (Anderson, 2014). Within disability studies, women with disabilities are often identified as a vulnerable population (Scully, 2014)—a classification that, while highlighting structural disadvantage, may also reinforce dependency and undermine autonomy (Lajoie, 2018). Their experiences are shaped by the intersection of gender and disability, which generates multiple and compounding forms of marginalisation (Emmett and Alant, 2006).

### 1.1 Vulnerability and women with visual impairments in China

Globally, women with disabilities face double discrimination, both as women and as people with disabilities (Shakespeare, 2018). Educational exclusion remains significant: only 41% of girls with disabilities complete primary education compared with 52% of boys with disabilities (UNICEF, 2021). In healthcare, paternalistic and ableist assumptions restrict access to reproductive services, as women with disabilities are often perceived as asexual or unfit for motherhood (O’Reilly, 2003). In the labour market, they are paid less and have fewer career opportunities than men with disabilities and women without disabilities (O’Hara, 2004). Women with disabilities are three times less likely to be employed in developed countries (WHO, 2011), and up to 80% remain unemployed in developing regions (United Nations, 2018). They also experience sexual violence 1.5–3 times more frequently than women without disabilities due to social isolation, dependence, and inadequate legal protection (Brownridge, 2006; Hughes et al., 2012).

In China, the ratification of the United Nations Convention on the Rights of Persons with Disabilities in 2008 marked progress in disability rights (Petersen, 2010). However, women with disabilities continue to experience structural inequality in education and employment. The education system remains dominated by special schools, with limited academic pathways. Gender norms further disadvantage them, as families often prioritise the education of children without disabilities (Guo, 2017). In employment, women with disabilities have lower participation rates and are largely confined to low-paid, gendered sectors such as massage work (Wang and Li, 2018; Xiong and Liu, 2023). Despite policy efforts to promote economic inclusion, many remain financially dependent on their families (Wang and Li, 2018). Cultural expectations surrounding marriage and caregiving reinforce these inequalities, as women with disabilities face stigma in the marriage market and struggle to balance employment with domestic responsibilities (Chen, 2019; Xiong and Chen, 2024).

For women with visual impairments, vulnerability is further intensified by occupational segregation and restricted educational access. Since China’s economic reforms in 1978, state policy has promoted vocational self-sufficiency for people with disabilities (Qu, 2022). Yet for those with visual impairments, opportunities have been narrowly channelled into massage work, a field culturally linked to the belief that blind individuals possess superior tactile sensitivity (China Disabled Persons’ Federation, hereafter CDPF, 1997). The CDPF institutionalised this pathway by establishing massage training schools and massage parlours; in 2023, 21,363 people with visual impairments received massage training, while data on alternative careers were absent (CDPF, 2023). The education system reinforces this pattern: most students with visual impairments access higher education through special entrance exams that offer only massage or music majors, despite the introduction of the Braille *Gaokao* – the National College Entrance Examination*—*in 2014 (Chen, 2025; Guo, 2017; Ma, 2014; Ma and Ni, 2020). Within the labour market, China’s massage industry is disproportionately populated by women with visual impairments, a pattern shaped less by individual preference than by policy-driven employment support arrangements (Lu et al., 2025). In addition to occupational channelling, women with visual impairments face gendered disadvantages, including restricted career options, workplace discrimination, and heightened risks of harassment, particularly in small massage parlours where oversight is limited (Xiong and Liu, 2023). Marriage often becomes a perceived route to stability, yet gendered expectations and caregiving burdens continue to constrain autonomy and social participation (Xiong and Chen, 2024).

### 1.2 Theoretical framework: vulnerability

#### 1.2.1 Vulnerability theories

Vulnerability arises when a person lacks control over events that threaten what matters to them (Anderson, 2014). It is commonly associated with adverse situations—events that individuals experience as undesirable or that are socially construed as such (Scully, 2014). As such, vulnerability is closely tied to power relations. When individuals gain greater control over the forces shaping their lives, vulnerability is reduced; when such control is diminished, vulnerability intensifies (Anderson, 2014). In this sense, pandemics such as COVID-19 have had profound and disproportionate impacts on people with disabilities, as pre-existing social injustices and discriminatory structures have exacerbated vulnerability and constrained access to protection and support (Scully, 2020).

A broader, sociopolitical conceptualization emerges in Butler’s (2004) theory of precarity, which reframes vulnerability as a structurally distributed condition rather than an individual deficit. Butler (2004, 2016) argues precarity is embedded in the social fabric—shaped by societal and structural inequalities that leave some groups more exposed to suffering. That precarity is not universal but differentially allocated through normative frameworks that determine whose lives are grievable and whose are disposable. Precarity operates at the intersection of bodily dependence—the inherent need for care, assistance, and social support that sustains life; social recognition—the extent to which individuals are acknowledged as valuable, legitimate members of society; and state regulation—the institutional and policy frameworks that allocate protection, rights, and welfare. When these interdependent dimensions fail to provide adequate support, certain populations (e.g., women and people with disabilities) become systematically exposed to harm, exploitation, and exclusion (Butler, 2004). This framework can explain how, in China, the interplay of gender norms, disability stigma, and welfare exclusions produces forms of structurally induced precarity for women with visual impairments. In Frames of War (Butler, 2016), Butler posits that precarity is politically induced: neoliberal governance, labour deregulation, and welfare retrenchment deliberately produce precarious subjects whose survival depends on precarious work, conditional aid, and familial obligation.

Critics, however, highlight the abstraction and universality of Butler’s account. Fraser (2013) and McNay (2014) argue that it underplays material hierarchies of class, gender, and race, whereas disability theorists such as Goodley (2014) and McRuer (2018) caution that framing dependence as vulnerability risks reinscribing able-bodied norms. Integrative perspectives, such as Kafer (2013), call for embedding Butler’s ethical ontology of vulnerability within intersectional and material analyses of capitalism, patriarchy, and ableism.

#### 1.2.2 Vulnerability and disability

Scully (2014) further distinguishes between two types of vulnerability related to disability: inherent and contingent vulnerabilities. Inherent vulnerabilities stem from an individual’s physiological or biological traits, such as physical weakness or chronic pain. While social environments can be adjusted to accommodate these vulnerabilities, it is impossible to eliminate them through such changes (Scully, 2014). In contrast, contingent vulnerabilities arise directly from the social environment and are shaped by social factors that create and sustain situations of vulnerability (Scully, 2014). For instance, if a workplace is not accessible for a person in a wheelchair, that individual must rely on someone else for assistance to navigate the space. This dependency creates vulnerability, as the person in the wheelchair is subject to the other individual’s actions and may face potential exploitation of that power. In this context, inherent vulnerability linked to biological traits aligns with the medical model (Llewellyn and Hogan, 2000), which views disability as a medical issue. On the other hand, contingent vulnerability can be understood through the social model (Oliver, 2018), where vulnerability depends on social barriers, such as an inaccessible environment.

Perceptions of vulnerability are closely tied to issues of autonomy and the recognition or denial of legal capacity (Arstein-Kerslake, 2019). Women with disabilities or people with disabilities, in general, are often disproportionately categorised as inherently vulnerable (Butler, 2016), leading to the perception that they require more protection than other groups. This has resulted in a loss of autonomy across various contexts. The denial of autonomy exacerbates contingent vulnerability, frequently misinterpreted as an inherent vulnerability. This creates a cyclical pattern of labelling, reinforcing the idea that women with disabilities or people with disabilities generally need special protection and are incapable of making independent decisions or exercising legal rights on par with others (Arstein-Kerslake, 2019). However, recognising the agency and autonomy of women with disability is a critical step toward breaking this cycle and reducing both inherent and contingent vulnerabilities. In line with Savaş et al. (2023), the analysis of vulnerability also requires attention to empowerment. The authors argue that vulnerability and empowerment are interlinked: while vulnerability may seem to signify powerlessness, it also creates opportunities for collective action and resistance.

### 1.3 Overview of the present research

Although the vulnerability of women with disabilities has been widely discussed, most studies still approach gender and disability as separate axes of inequality (Emmett and Alant, 2006; Shakespeare, 2018). In China, existing research tends to focus either on disability employment policy (Wang and Li, 2018; Qu, 2022) or on gender inequality in education and labour markets (Guo, 2017; Chen, 2019), with limited attention to their intersection. Studies specifically addressing women with visual impairments remain sparse, often describing educational and employment barriers in isolation rather than analysing how these structural forces interact to reproduce disadvantage.

Few investigations employ vulnerability theory to explain how education and employment systems create and sustain dependence and restricted autonomy. While national statistics reveal low participation rates in both schooling and work among people with disabilities (CDPF, 2023), the underlying mechanisms—such as gendered expectations, ableist norms, and institutional constraints—remain underexplored.

This study addresses these gaps by examining how intersecting barriers in education and employment shape compounded vulnerabilities among the women with visual impairments interviewed in this study, situated within particular institutional contexts in China. Drawing on Butler’s (2004, 2016) concept of precarity and Scully’s (2014) distinction between inherent and contingent vulnerability, it explores how social, cultural, and policy contexts transform impairment into structural precarity while simultaneously revealing spaces of resistance and agency. The study contributes to understanding how gender and disability jointly structure inequality and offers insights for advancing inclusive education, equitable employment, and social recognition.

## 2 Methodology

This study explored how barriers to education and employment create and compound vulnerability among women with visual impairments within specific institutional and social contexts in China. Seven women aged 25–40 years (See Table 1), all with formal or informal work experience, were purposively selected from a larger project involving 26 individuals with visual impairments; the remaining 19 participants were men and thus excluded from this gender-focused analysis. The age cohort was chosen to ensure participants were born after the 1978 economic reforms and reached school age during the gradual expansion of disability services following the establishment of the CDPF in 1988. All participants were legally blind at the time of the interviews, though two reported progressive vision loss during childhood. Four participants originated from northern provinces and three from southern provinces; at the time of study, all resided in urban centres, but four were born in rural counties.

**Table 1:** Participants’ basic information.

Case	Age range	Childhood location type	Current visual impairment level	Education level completed	Type of work	Marital status	Parental status
Juan	30–40	Rural	No vision	Massage vocational secondary school	Massage	Married	Yes
Le	20–30	Rural	No vision	No formal education experience	Massage	Married	Yes
Lili	20–30	Urban	No vision	Bachelor’s degree in piano course	Piano tuning	Single	No
Ling	30–40	Rural	No vision	Massage vocational secondary school	Massage	Partner	No
Lisha	30–40	Rural	No vision	Dropped out during special primary school	Massage	Married	Yes
Tina	30–40	Urban	No vision	Bachelor’s degree in massage course	Massage/teaching massage	Single	No
Kaili	20–30	Urban	No vision	Junior college in piano course	Library	Single	No

Data were collected through in-depth semi-structured interviews conducted in Mandarin by the author in 2022. Due to COVID-19 mobility restrictions, all interviews were conducted remotely via telephone, lasting approximately 2 h each. Participants were recruited purposively via a disability organization in Henan province and through snowballing to meet the inclusion criteria (female, with work experience). Interview questions focused on educational experiences, occupational trajectories, family responsibilities, welfare access, and social barriers. Interviews were audio-recorded with consent and transcribed verbatim in Chinese.

Data collection ceased after seven interviews, as thematic saturation was reached. Core themes—including educational gatekeeping, vocational channelling, rural–urban disparities, occupational stereotyping, and harassment—were consistently reflected across participants, with the final interview serving as a negative case that confirmed rather than contradicted these patterns. This convergence, across regional, educational, and socio-economic backgrounds, indicated that additional female participants would likely yield redundant information within this bounded scope, prioritizing analytical depth over breadth (O’Reilly and Parker, 2013).

Analysis followed Braun and Clarke’s (2006, 2021) thematic analysis framework to explore both explicit and implicit meanings in participants’ narratives, focusing on how educational and occupational barriers produce vulnerability. Analysis was conducted on original Chinese transcripts to preserve linguistic and cultural nuances; salient excerpts were translated into English and back-translated to ensure fidelity (Squires, 2009). Four superordinate themes were generated: (a) Educational gatekeeping and delayed personhood, (b) Occupational channelling and economic dependence, (c) Gendered familial obligation, and (d) Intersectional invisibility within policy structures. Theme credibility was enhanced through peer debriefing with two independent qualitative researchers, member checking with three participants, and reflexive memo-writing throughout the analysis.

The studies involving humans were approved by the Research Ethics Committee at the School of Sociology and Social Policy, University of Nottingham. All procedures—including informed consent, confidentiality, and COVID-19 safety measures—were rigorously followed. These themes directly informed the structure of the Findings section and guided the theoretical interpretation in the Discussion, ensuring conceptual coherence between data, analysis, and argumentation.

## 3 Findings

Across the educational and work trajectories of the participants, a consistent pattern of systemic exclusion and constrained opportunities emerged. None of the women participants had attended preschool, despite preschool enrolment being a near-universal milestone for children without disabilities in China, with national gross enrolment rates reaching 88.1% in 2021 (92.2% in urban areas) (National Bureau of Statistics of China, 2022). The absence of early educational access illustrates how social and institutional arrangements produce contingent vulnerability, limiting autonomy even before formal schooling begins.

For most participants, formal education began at the primary level within segregated special schools for the blind, with Le being the only participant who reported having no formal educational experience. Drawing on the broader dataset, which included interviews with 19 men with visual impairments, similar trajectories of exclusion from mainstream preschool and schooling were evident across gender, regardless of rural or urban origin. Vocational stereotyping—most notably the association between visual impairment and massage work—also emerged as a shared feature of educational and occupational pathways (see Chen, 2025 for a detailed analysis). However, while vocational channelling into massage constituted a common structural pathway for both women and men with visual impairments, its consequences were unevenly distributed. Participants described that girls and women with visual impairments faced lower rates of educational access and were exposed to additional gender-specific risks, including sexual harassment in both educational settings and massage workplaces. These experiences indicate that gender did not alter the existence of vocational channelling itself, but shaped differential exposure to risk and constraint across the life course.

### 3.1 Barriers to and at education

#### 3.1.1 Limited access to education

As mainstream schools rejected most participants, nearly all attended special education schools, highlighting systemic barriers that produced early contingent vulnerabilities (Scully, 2014). Ling persistently sought enrolment in a local preschool and repeatedly told her parents, “I must go to school,” but faced rejection due to the lack of adapted entry assessments. She vividly recounted her failed preschool registration:

…. The principal wrote a few words on the blackboard. At that time, I could see clearly, because he wrote big, and his written words had colour [like rainbow writing]. Because of this, I could see clearly. Then he handed me a book, and he said, “Can you read the writing on it?” When I read it, oh no, I couldn't see it totally. He said, “That won't work, you can't read the words in the book clearly, that won't work”. Then we had no way. I didn't believe the result, but there was no way. (Ling)

The preschool registration system was designed primarily for children without disabilities, meaning that visual impairments were treated as exceptions or deficits. The absence of accessible assessments demonstrates structural mechanisms that constrain autonomy, reinforcing the educational system itself as a source of vulnerability. Nevertheless, Ling’s persistent efforts to attend school, despite repeated rejections, illustrate early agency in negotiating structural barriers, signaling her potential for empowerment—a dynamic that will be further analysed through Butler’s (2004) precarity and Scully’s (2014) contingent vulnerability framework in 4.2.

Access to schools for the blind, although formally available, was limited and frequently required long-distance travel. Regardless of whether participants were born in urban or rural areas, all reported the need to travel substantial distances to attend such schools, as schools for the blind were typically established at the provincial rather than city level. As Lili explained:

"The only school for the blind in my province was hundreds of kilometres away. I had to board there and travel long distances regularly." (Lili)

Similarly, Lisha’s primary school was 500 kilometres from her home. Ling experienced a 2-year wait for a new class of students with visual impairments, and Kaili was restricted by household registration policies from attending a school for the blind in another city. These experiences demonstrate how geographic and bureaucratic barriers create structural precarity (Butler, 2004), making access to education contingent upon economic resources and family support. Such constraints illustrate how contingent vulnerability interacts with inherent vulnerability (Scully, 2014), as physical impairment intersects with structural and institutional exclusions.

#### 3.1.2 Being treated as a patient

Early medicalisation often displaced opportunities for educational and social development, particularly for those participants (Lili, Tina, and Kaili) who were born in urban areas and grew up in relatively advantaged financial conditions. Instead of entering the preschool, Tina and Lili continually saw doctors seeking treatment for their eye conditions. Tina, who was partially sighted and now with no vision, said she seemed to live in the hospital when she was a kid. When talking about her experiences with eye treatments at that age, Tina kept repeating, “I had no childhood”:

When I was young, I actually had a rough life before I went to school. I had my eyes checked every day, and I went to the hospital every day. I felt that I lived in the hospital when I was a kid. I felt that when I saw the current children, I didn't have a childhood. That's true. I didn’t have a childhood when I was a child. I think I was 3 or 4 years old. Ever since I can remember, I have felt that I was very precocious… I knew that my family [pause] worked hard to earn money, and then after making money, they took me to the hospital. Then after going to the hospital, they started to work hard to earn money again, and then after this, I went to the hospital again. It went on and on like this until I went to [primary] school. (Tina)

Routine medical interventions limited autonomy, framing her as a medical subject rather than an individual capable of agency. Similarly, Lili was told by a doctor that she “should never have been born,” reflecting deeply entrenched ableist medical attitudes. Experiences such as these exemplify the interplay between inherent vulnerability (Scully, 2014), arising from biological traits, and contingent vulnerability (Scully, 2014), arising from social practices that restrict autonomy and participation. These medicalized childhood experiences set a precedent for later structural exclusions in education and work, demonstrating early precarity in life trajectories.

#### 3.1.3 Parental beliefs and educational exclusion

Parental attitudes reinforced systemic barriers. Three participants (Lisha, Le, and Ling) reported that their parents believed formal education was not worth pursuing for children with visual impairments, viewing it as a waste of time compared to practical skills like massage. This attitude influenced Lisha and Le’s decisions to abandon formal education and pursue massage training instead. Lisha, for example, was not allowed to continue her education beyond Grade 2 due to her father’s patriarchal beliefs, which prioritised skills that would enable her to support herself in the future rather than completing her compulsory education. This decision, although deeply hurtful to Lisha, reflects a common belief in certain communities that education for girls, particularly girls with disabilities, is unnecessary or impractical (Zhou, 2012):

To be honest, my father preferred sons to daughters, and I was also disabled, so he didn't take me seriously… In the second year [Grade 2 in special primary school], my father didn't want me to continue studying. What he meant, that is, I was a girl and a disabled person. When I went to school, I should learn something practical, then earn money, and support myself. He said that I went to school, but I didn't learn anything, and I didn't know how to do housework either, or I was not able to earn money [in the special school] … So, he didn't want to send me to school. But as I said just now, I felt that I couldn't stand it [staying at home] even though I only spent the winter and summer vacations at home. If I stopped going to school, I was going to break down. I didn't know what I should do, or what I would become in the future. I was very anxious, but no one cared about me. At that time [in 2000], there was no telephone, and it was impossible to contact the outside world at any time by WeChat [Chinese social media] like now. Finally, I backed down, and I told my dad that I would not follow this normal route [educational trajectory] to go to school like elementary and junior high school. I just had to give up. (Lisha)

Lisha’s father’s view toward a girl with visual impairments reflects compounded vulnerability caused by gender and disability. On the one hand, Lisha faced obstacles to accessing formal education because it was considered useless for a child with disabilities to go to school; on the other hand, she was not taken seriously and cared for well by her family as a daughter. Her two intersecting identities made her father not allow her to continue going to school. Her experience illustrates how vulnerability was produced through the intertwined effects of disability and gender, with discrimination and marginalisation mutually reinforcing one another and shaping her life trajectory (Davis, 2008).

Similarly, Le’s belief that she was “useless” due to her disability and gender, instilled by her parents. Le internalised the perception of being “useless,” reflecting the personal and social construction of vulnerability. These examples illustrate contingent vulnerability (Scully, 2014) at the family level, where social norms and expectations intersect with gendered assumptions about disability to restrict access to educational opportunities. The intersection of gender and disability intensified vulnerability, limiting participants’ capacity to challenge exclusionary decisions and reinforcing structural precarity in both educational and later occupational domains.

However, Kaili, who was born into relatively advantaged families, reported different experiences. Kaili’s mother believed that her daughter could develop skills beyond massage and therefore arranged private lessons in piano and English during childhood. Distrusting the educational quality of the school for the blind, she withdrew Kaili from special schooling for several years and instead provided home-based education.

#### 3.1.4 Targeted vocational education

Vocational training, particularly massage, was introduced in primary school and emphasized throughout compulsory, secondary, and tertiary education. Ling and Haidi mentioned having massage courses as early as Grade 5 or 6 in their special primary schools. The current academic literature discusses little about when students with visual impairments are expected to engage in massage-related activities, but Ling explained this situation:

When I was in primary school, because [the school] first considered the fact that many of us were [relatively] older students, [the school] also wanted us to enter society [get employed in the labour market] as soon as possible. (Ling)

Different admission ages influenced which courses students received, with massage training positioned as a practical route into the labour market. This early vocational focus reflected prevailing assumptions that people with visual impairments were best suited for caregiving or manual roles, while simultaneously narrowing educational trajectories at an early stage. Rather than supporting exploration or skill diversification, these arrangements set students on predetermined pathways, shaping future options long before meaningful choice became possible. In this sense, early vocationalisation exemplifies contingent vulnerability, as institutional decisions—not individual preferences—played a decisive role in structuring educational and occupational futures (Scully, 2014).

Autonomy was further constrained at the level of tertiary education. Tina and Lili reported that only a limited number of universities offered specialised entrance examinations for students with visual impairments, and that available programmes were largely confined to massage or music (Ma, 2014; Ma and Ni, 2023; Zhang, 2021). These higher-education gatekeeping practices did not merely channel students into particular fields but also imposed an upper boundary on aspiration, qualification, and mobility. Such institutional constraints illustrate how precarity is reproduced through restricted access to recognised credentials, with long-term implications for employment security and social positioning (Butler, 2004).

### 3.2 Barriers to and at work

#### 3.2.1 The cultural stereotype that “the blind should learn massage”

More than half of the participants reported that social pressures—from family, friends, and local communities—played a significant role in their decision to pursue massage training. Le’s experience illustrates this dynamic: Despite having no formal education, she was introduced to massage training by a fellow villager who had seen a person with visual impairment working in a massage parlour. Her story highlights how individuals with visual impairments are often funnelled into specific career paths, reflecting both the limited employment options available and the lack of autonomy in career choices for people with disabilities.

Lili’s experience further underscores the weight of family expectations. Despite her deep-rooted passion for playing the piano, her family pressured her into massage work, reinforcing the gendered and economic vulnerabilities faced by women with disabilities. The assumption that people with visual impairments are best suited for massage not only constrained her choices but also exemplified how cultural norms and familial expectations can override individual aspirations, leaving limited room for alternative career paths.

#### 3.2.2 Barriers to integration into the workplace

Lisha and Le’s experiences illustrate that not all massage parlours, despite being seen as an industry where people with visual impairments could thrive, are barrier-free. In large massage parlours, they faced considerable challenges, such as difficulty identifying clients and rooms, and struggled to integrate with colleagues without disability. Lisha described feeling isolated as the only employee with visual impairment, which compounded her vulnerability in the workplace. Moreover, the inaccessible work environment—including issues like travelling (the accommodation provided was outside the massage parlour)—led Lisha to leave a job where she faced social and logistical barriers. These working experiences highlight how the workplace environment itself is structured in ways that exclude employees with disability. Despite the expectation that massage parlours should be accommodating, they often fail to provide basic accessibility features, which leads to contingent vulnerability (Scully, 2014). Moreover, Lisha mentioned that massage work might not be a suitable job for women with visual impairments, as females are more likely to have less body strength than men, and their massage effect is at a relatively disadvantaged level.

On the other hand, Lili and Kaili, who has the chance to learn piano during their childhood, encountered another vocational stereotype for people with visual impairments—the creative industries, particularly music. Their experiences highlight systemic challenges within this field. Lili aspired to be a piano teacher and studied piano for 20 years, eventually graduating from a special college. However, when she sought employment at a local piano shop alongside a classmate with low vision, she was rejected. Unlike Lili, her classmate could travel and work independently, while Lili, who was completely blind, required assistance. Her reliance on her grandmother as a personal assistant gave employers the impression that she lacked independence, leading to their decision not to hire her. This shows that employers in the music industry often hesitate to hire women with visual impairments due to perceived travel difficulties, further limiting their employment opportunities. Workplace inaccessibility and lack of accommodations produced structural barriers that heightened vulnerability and limited independence. Such conditions exemplify precarity in the workforce, where institutional arrangements constrain employment options and reinforce societal assumptions of incapacity (Butler, 2004).

#### 3.2.3 Barriers to seeking help when working at risk

Sexual harassment emerged as a significant workplace and educational risk. Four participants (Le, Lili, Ling, and Lisha) mentioned that sexual harassment happened to them, either in special schools or in massage parlours. For three of the five female participants who worked in massage parlours, experiences of sexual harassment were frequent, often involving male clients engaging in inappropriate physical contact:

Many [male customers] like to do some small movements. [For example] he [one of these male customers] lies on the [massage bed]… When we [the female masseuse with visual impairments] massaged him, we had to bend over… Then, he may subconsciously reach out and touch your leg or reach out and touch your breast. He liked to do these small movements. Then we didn’t want such people to come. [When] it was our turn [female masseuse’s turn to provide massage services for these people], we may say the next [male colleague will do it]. And because of this, we were punished by the boss… Generally, the boss would only focus on money [profit]; they would not consider these things [sexual harassment of female employees by customers]. (Ling)

It definitely wouldn't develop to the point where he really did something to me. When we [female massage workers with visual impairments] were working [in other massage parlours] [and] encountered this kind of customer [touching us], we would usually say - I'm not doing this; please respect me. If he continued to do something [keep touching us], I would say - I'm sorry, please pay the bill; I won't [offer] massage [services] anymore. Then I would go to the reception and tell the boss [this service ended], and usually this kind of customer would pay the bill, and then I would leave [and wait for the next customer]. (Le)

Ling’s experience of being punished by the boss for refusing inappropriate customers illustrates the power dynamics at play, where workers’ safety and dignity are often subordinated to economic goals. Additionally, Le’s response to harassment, where she asserted her boundaries by refusing to continue the massage and informing the boss, shows an attempt to regain some degree of agency in a difficult situation. However, the fact that she had to act independently without institutional support reveals the lack of protection for women with disabilities in the workplace. More importantly, Le also said sexual harassment is not only caused by the clients but also sometimes by her male colleagues with visual impairments. None of this has ever come to light previously. This suggests that sexual harassment is not confined to interactions with people without disabilities but can also stem from within the disabled community itself, where gendered power imbalances and internalised stereotypes play a role in perpetuating harmful behaviours.

Lisha indicated that harassment largely ended only after establishing her own business with her husband. These experiences highlight how economic dependence, lack of protective structures, and gendered dynamics converge to create structural precarity and reinforce contingent vulnerability (Scully, 2014; Mustaniemi-Laakso et al., 2023). These patterns also underscore the importance of autonomy, self-advocacy, and ownership over work environments as protective factors against exploitation.

Overall, the findings suggest that the women participants in this study experienced multilayered barriers rooted in cultural norms, institutional constraints, and gendered expectations within specific institutional contexts in China. These intersecting forces shape their educational and employment trajectories, reinforcing structural inequalities and limiting agency. While participants demonstrated resilience and adaptability within these constraints, their narratives underscore the systemic nature of vulnerability rather than individual deficiency. The following discussion situates these findings within existing scholarship and theoretical frameworks to deepen understanding of how gender, disability, and social structures intersect to produce and sustain vulnerability.

## 4 Discussions

### 4.1 Comparison of prior research

The findings of this study align with, and add depth to, the expanding body of literature examining the intersectional disadvantages faced by women with disabilities. Globally, women with disabilities experience what Shakespeare (2018) terms “double discrimination,” a form of compounded marginalisation produced by the interaction of gender and disability. Educational exclusion remains one of the most persistent dimensions of this inequality. As reported by the UNICEF (2021), girls with disabilities are less likely than boys with disabilities to complete even basic education, reflecting deeply rooted inequities in access and participation.

In the Chinese context, prior studies have documented limited educational opportunities for learners with visual impairments, particularly within mainstream schooling, where systemic and attitudinal barriers persist (Ma, 2014; Zhang, 2021). The continued reliance on segregated schooling models and narrowly vocational curricula—most notably in massage or music—has further constrained students’ academic and occupational horizons. Building on this literature, the present study confirms these patterns while adding nuance by demonstrating how such institutional arrangements intersect with gendered expectations and policy priorities to intensify vulnerability. Women with visual impairments navigate not only educational exclusion but also patriarchal norms that frame them as dependent, fragile, or unsuitable for professional advancement. Within this context, parental beliefs function as a key mediating site through which employment-oriented policy logics—most notably the policy-driven concentration of women with visual impairments in massage work (Lu et al., 2025)—filter into family-mediated educational decisions.

At the same time, the study demonstrates that parental expectations varied according to family resources and interpretations of what constituted a viable future. Among participants from less advantaged families, concerns about economic survival and social respectability often led formal education to be viewed as impractical or wasteful when vocational routes such as massage were institutionally promoted as secure and legitimate pathways. In more economically advantaged families, parents were more willing to invest in extended education or skill diversification—such as piano or language training—but such support remained conditional, bounded by assumptions about long-term economic viability and dominant norms of “realistic” achievement.

Employment remains another critical arena where gender and disability intersect to produce structural disadvantage. Previous research has emphasized the overrepresentation of people with visual impairments in the massage industry, a pattern reinforced by historical state policy and cultural narratives that frame this profession as both respectable and “suitable” for the blind (CDPF, 1997; Wang and Li, 2018). While such channels provide a degree of economic stability, they also confine individuals within narrowly defined occupational spaces. The findings of this study reveal that women with visual impairments experience these constraints more acutely, facing not only limited vocational choice but also gendered inequalities within workplaces, including harassment and exclusion from professional networks.

Compared with earlier scholarship (e.g., Xiong and Liu, 2023; Ma and Ni, 2020), this study extends understanding by demonstrating that structural and cultural forces operate simultaneously to delimit agency. Gendered expectations compound disability-related stigma, producing a distinct form of intersectional precarity that affects both professional integration and personal autonomy. These findings underscore the necessity of intersectional analyses that move beyond single-axis approaches to disability and gender, recognizing the multiplicative—not additive—nature of social disadvantage.

### 4.2 Theoretical and practical implications

The findings can be interpreted through Butler’s theory of precarity and Scully’s distinction between inherent and contingent vulnerability, which together illuminate how the women participants in this study, situated within specific institutional contexts in China, experience multilayered and institutionally produced vulnerability. Butler (2004, 2016) conceptualises precarity as a structurally distributed condition rather than an individual weakness. Certain lives, she argues, are rendered more “precarious” when social and institutional frameworks fail to provide recognition, protection, and support. In the Chinese context, precarity is sustained by intersecting systems of gendered expectations, disability stigma, and policy frameworks that prioritise labour utility over social inclusion. Participants’ narratives reveal how early vocationalisation—such as the introduction of massage training in primary school—positions students with visual impairments as future labourers rather than autonomous learners. This is consistent with the state’s broader approach to disability employment, where policy documents like the Law on the Protection of Persons with Disabilities (Standing Committee of the National People’s Congress, 1990, 2008) and the Regulations on the Education of Persons with Disabilities (Ministry of Education of the People’s Republic of China, 2017) promote vocational self-reliance as a one of the key goals. While well-intentioned, this emphasis channels students into narrow occupational pathways, reflecting what Butler (2016) calls normative frames of livability—social scripts that define which forms of existence are recognised as valuable.

The educational and employment systems, therefore, do not merely reflect existing inequalities but actively produce precarity. Early vocational training limited tertiary education options, and the dominance of massage as an occupational route institutionalised dependency under the guise of empowerment. These findings show that precarity is politically induced: social and policy structures organise who is exposed to risk, who is protected, and who remains invisible within mainstream education and labour markets. Such dynamics echo Butler’s argument that precarity is differentially allocated, with women with visual impairments occupying a position of compounded marginality.

Scully’s (2014) distinction between inherent and contingent vulnerability provides an additional analytic layer. While visual impairment itself constitutes an inherent vulnerability arising from biological or sensory differences, the more consequential vulnerabilities observed in this study are contingent—socially produced through exclusionary institutions and cultural assumptions. Restricted access to mainstream schooling, gender-biased parental decisions, workplace discrimination, and sexual harassment exemplify such contingent vulnerabilities. However, as several participants indicated, these are frequently misinterpreted as inevitable consequences of impairment. The conflation of inherent and contingent vulnerability results in paternalistic overprotection, policy infantilisation, and societal expectations that women with visual impairments require constant supervision and cannot exercise independent agency. This misrecognition, as Butler (2004) notes, reinforces precarity by naturalising structural disadvantage and masking its political origins.

The integration of Butler’s and Scully’s perspectives underscores that vulnerability is both embodied and socially mediated. It operates at the intersection of the biological and the political, revealing how systemic exclusion translates into lived fragility. Yet, as Savas et al. (2023) argue, vulnerability can also be a generative force—an entry point for collective action and empowerment. Several participants expressed a desire to pursue education beyond massage training, and family investments in non-massage skills development indicate that agency persists even within structurally constrained systems. Recognising this dual nature of vulnerability—both limiting and enabling—shifts the analytic focus from protection to empowerment.

Practically, this conceptual synthesis points to several directions for intervention across education, employment, and welfare domains. From a policy perspective, addressing contingent vulnerabilities requires structural change that operates across these interconnected fields. In education, reforms should expand inclusive pathways by strengthening the implementation of the Braille Gaokao and enabling access to a wider range of tertiary programmes beyond narrowly channelled options such as massage or music.

At the same time, the findings call for critical reflection on the timing and rigidity of early vocational training for students with visual impairments. While vocational education can provide valuable skills, its premature introduction—particularly when presented as the primary or only viable pathway—risks foreclosing later educational choices. Policies should therefore avoid early, irreversible vocational channelling and instead support flexible educational trajectories that allow students to pursue general education alongside vocational exploration, preserving access to tertiary education at later stages. Within welfare systems, support should move beyond subsistence provision to facilitate educational continuity, skills development, and transitions into diverse forms of employment.

At the level of employment, more specific and targeted measures are required to address the gendered risks identified in this study. Given the concentration of women with visual impairments in massage work, labour regulations should mandate clear workplace safety standards and enforce anti-harassment policies, particularly in small massage parlours where oversight is limited. Accessible reporting mechanisms, independent complaint channels, and regular inspections are essential to reduce everyday exposure to harassment and abuse. In addition, legal aid and rights-based support must be made available to women with visual impairments, including accessible legal information, disability-sensitive legal assistance, and protection from retaliation.

The findings also suggest implications for parental engagement in educational and employment decision-making. Rather than framing parental choices as individual attitudes, policy interventions should recognise families as operating within constrained institutional and policy environments. Providing accessible information, counselling, and guidance for parents of children with visual impairments—particularly regarding long-term educational options beyond early vocational routes—may help counter the perception that narrow occupational pathways are the only realistic futures available.

Alongside regulatory measures, promoting social recognition remains central to mitigating precarity. Public awareness campaigns, inclusive teacher training, and media representation can challenge the ableist and gendered stereotypes that underpin both occupational channelling and workplace harassment. In this sense, vulnerability is not an individual burden but a social responsibility, calling for institutional arrangements that redistribute power and protection. By situating women with visual impairments within frameworks of precarity and vulnerability, this study highlights how educational systems, labour governance, and welfare provision jointly shape both constraint and possibility.

### 4.3 Limitations and future directions

This study has several limitations that should be acknowledged. The empirical material is drawn from a small qualitative sample of women with visual impairments who experienced particular educational and occupational trajectories within specific institutional contexts in China. As a qualitative study, the findings are contextually grounded and intended to illuminate patterns and processes rather than to claim statistical representativeness across all women with visual impairments in China. Future research could incorporate larger and more diverse samples, including comparative analysis across gender, region, and rural–urban backgrounds, to further examine how vulnerability is differentially produced. The analysis focuses primarily on education and employment, leaving other domains—such as health, family relations, and civic participation—less fully explored. Extending empirical attention to these areas would enable a more holistic understanding of vulnerability across the life course. In addition, the cross-sectional design captures participants’ experiences at a particular moment in time. Longitudinal research could more fully examine how vulnerabilities evolve over the life course, particularly as women with visual impairments transition into adulthood, navigate marriage and caregiving roles, and engage with employment systems.

Overall, this study contributes to disability research by highlighting the compound vulnerabilities experienced by some women with visual impairments within specific institutional and social contexts in China, and by demonstrating how structural, cultural, and personal factors intersect to shape these experiences. By integrating theoretical and empirical analysis, the study offers context-sensitive insights that speak to ongoing debates on educational access, occupational inclusion, and gendered vulnerability, while also informing policy and practice aimed at reducing inequality and enhancing protection for women with disabilities.

## Data Availability

The original contributions presented in the study are included in the article/supplementary material, further inquiries can be directed to the corresponding author.
